# Peripherally inserted central catheters can be an alternative to tunneled central venous catheters in chemotherapy for hematological and oncological pediatric patients

**DOI:** 10.1007/s00383-023-05545-4

**Published:** 2023-09-06

**Authors:** Yuko Kamata, Yuki Mizuno, Kentaro Okamoto, Shota Okamoto, Yoshifumi Ito, Aya Nishigata

**Affiliations:** Department of Pediatric Surgery, Tokyo Medical and Dental University Hospital, 1-5-45, Yushima, Bunkyo-ku, Tokyo, 113-8519 Japan

**Keywords:** Peripherally inserted central catheter, Chemotherapy, Pediatric, Tunneled central venous catheter

## Abstract

**Purpose:**

Tunneled central venous catheters (TCVs) are commonly used for pediatric chemotherapy. Recently, peripherally inserted central catheters (PICCs) have been used instead. Although PICC has the advantages of simpler insertion and fewer severe complications, there is little information on the efficacy of PICC compared to TCV in pediatric chemotherapy.

**Methods:**

Patients, aged younger than 18 years, with primary malignancy who received chemotherapy with PICC or TCV at our institution from December 2007 to August 2022 were included in the study. We retrospectively compared PICC and TCV using medical records.

**Results:**

Within the observation period, 133 catheters (73 PICCs and 60 TCVs) were inserted. The median indwelling time was 99 days for PICCs and 182 days for TCVs, with TCVs being significantly longer (*p* < 0.001). There were no significant differences in the incidence of complications, such as infections, thrombosis, obstruction, or mechanical accidents. Comparing patients treated with PICC (PICC group) versus those with TCV (TCV group), the time from diagnosis to insertion was significantly shorter in the PICC group (*p* < 0.001). In the PICC group, none of the patients required general anesthesia, and chemotherapy was completed with PICC only.

**Conclusion:**

PICC can be an alternative to TCV in pediatric chemotherapy.

## Background

Central venous catheters (CVCs) are commonly used to deliver chemotherapy for the management of hematologic and solid tumors in children. CVCs are particularly useful in children, who have a high burden of vascular puncture. CVCs allow for the reliable administration of highly tissue-damaging drugs and enable reverse blood draws for frequent blood tests, which are necessary for the chemotherapy of hematologic and solid tumors [1, 2]. However, pediatric patients with hematologic and solid tumors are at high risk for (1) infection and thrombosis due to abnormal blood cell counts and coagulation [3], as well as (2) complications related to CVCs due to frequent manipulations for the many drugs and blood transfusions required [4, 5]. Therefore, the choice of device for CVCs is important. CVCs include tunneled- or non-tunneled-central venous catheters (TCV or NTCV), peripherally inserted central catheters (PICC), and implanted venous-access ports (TIVAP). For pediatric patients requiring chemotherapy, TCVs are usually used. While TCVs have a lower catheter-related infection risk than NTCVs due to the creation of a subcutaneous tunnel [5, 6], general anesthesia is necessary for insertion, and sedation or local anesthesia is needed for removal because cuff is adhered to subcutaneous fat. Also, the procedure is associated with serious complications, such as pneumothorax and hemothorax, due to puncturing of the internal jugular vein and subclavian vein [7]. Overall, TCVs are burdensome for pediatric patients.

Recently, PICC has gained increasing attention as an alternative to TCV, due to its relative ease of insertion and safety. In particular, its usefulness in children has been reported in the literature [8, 9]. Since PICC is inserted through a peripheral vein, the risk of serious complications, such as pneumothorax or hemothorax, is negated. Also, since a subcutaneous tunnel is not necessary, general anesthesia is not required for its insertion and it can be easily removed by pediatricians. As such, PICC is considered ideal and less burdensome for pediatric patients as opposed to TCV. To date, few reports comparing PICC and TCV in children have been published. At our hospital, we used only TCVs for pediatric chemotherapy until 2015. In 2016, PICCs were introduced, and since 2018, PICCs have been used in almost all cases. In this retrospective study, we conducted a comparative analysis of the clinical factors to verify whether PICCs are superior to TCVs as a drug delivery route in pediatric patients with hematologic or oncological malignancies.

## Methods

### Study design

Patients, aged under 18 years of age, with primary malignancies who received chemotherapy without hematopoietic cell transplantation (HCT) with PICCs or TCVs at our institution from December 2007 to August 2022 were included in the study. Data, such as patient background, primary disease, catheter placement technique, and catheter-related complications, were obtained from the medical records. The patient background was matched by propensity score matching, and then, the data were re-analyzed for the matched groups. In addition, PICCs are classified into the open-ended type (O-PICCs) and the closed-ended type (C-PICCs). We also performed the analysis across the three groups: TCV, O-PICC, and C-PICC.

**Fig. 1:**
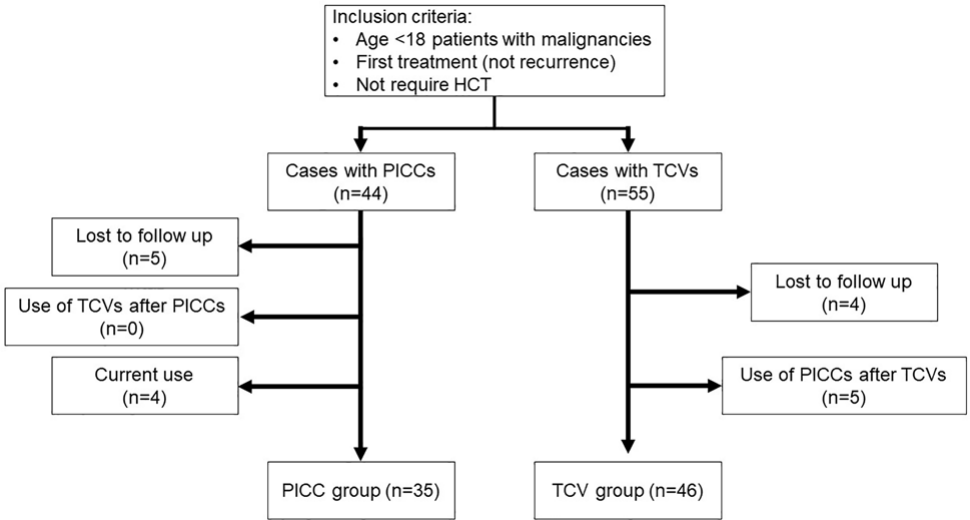
The schematic diagram of the study according to the case selection; *HCT hematopoietic cell transplantation, PICC* peripherally inserted central catheter, and *TCV* tunneled cuffed central venous catheter

Additionally, the group of patients who completed chemotherapy with PICCs only (PICC group) and the group of patients who completed chemotherapy with TCVs only (TCV group) were compared on a per-patient basis rather than on a per-catheter basis. Patients who were lost to follow-up, currently using a catheter, or who used both TCVs and PICCs during the course of their treatment were excluded. The analysis included 35 patients in the PICC group and 46 patients in the TCV group (Fig. 1). The number of days from diagnosis to the first catheter insertion, the total duration of treatment, the total number of catheters inserted, and the number of general anesthesia sessions were compared between the two groups.

Eligible patients were followed until they completed treatment and had the last catheter removed, were diagnosed with relapse, underwent HCT, died, or in August 2022, whichever came first. The study was approved by the ethics committee of our hospital (ethical approval number: M2022-190).

### Procedures

The decision whether to insert a single- or double-lumen catheter was determined according to the planned chemotherapy regimen. TCVs and PICCs were used to administer chemotherapy drugs, intravenous nutrition, and reverse blood sampling. TCV (Bard, Hickman 7Fr or Broviac 6.6Fr/4.2Fr/2.7Fr) was inserted in the supine position under general anesthesia.

PICC (Bard Access Systems Inc., Salt Lake City, UT, USA, Groshong 4Fr single lumen and 5Fr dual lumen or Cardinal Health, Argyle 3Fr single lumen and 4.5Fr dual lumen) insertion was performed under sedation or local anesthesia. Most PICCs were inserted by pediatric surgeons, but nine PICCs in the early stages of PICC introduction were inserted by pediatricians. The PICC was sutured at the puncture site if accidental removal was deemed likely. The catheter was stabilized by making a loop to prevent pulling and covered with a film dressing; in the case of the Groshong catheter, it was secured with a Statlock.

Catheters were flushed with heparinized saline once every two days when not in use, whether in the hospital or at home, as per international guidelines [10, 11]. Also, once a week, PICC and TCV puncture sites are disinfected and film dressings are changed.

### Statistical analysis

All statistical analyses were performed using SPSS software (version 22.0, IBM, Armonk, NY, USA). The Student’s t test, Mann–Whitney’s *U* test, and Pearson’s χ^2^ test were used for comparing between groups. Survival curves were drawn using the Kaplan–Meier method and compared by the log-rank test. To adjust for any differences in patient background between the groups, logistic regression analysis was performed to obtain a propensity score, and matching by propensity score was performed. A two-sided *P* value less than 0.05 was considered a statistically significant difference.

## Results

A total of 133 catheters including 73 PICCs (54.9%) and 60 TCVs (45.1%) were inserted during the observation period. Patient background details are shown in Table 1. The median age of the patients was 7.0 years (0–16 years) for PICCs and 4.0 years (0–17 years) for TCVs, respectively. The mean age of the patients with PICCs was significantly higher compared to that of patients with TCVs (*p* = 0.036). Patients with PICCs were significantly taller (*p* = 0.021) and heavier (*p* = 0.025) than those with TCVs.

**Table 1:** Baseline demographics and patient characteristics

	PICCs	TCVs	*P* value
	*n* = 73	*n* = 60	
Age, year, median (range)	7.0 (0–16)	4.0 (0–17)	0.036*
Male, *n* (%)	44 (60.3)	37 (61.7)	0.870
Height, cm, median (range)	122.1 (54.0–179.7)	105.8 (61.2–172.5)	0.021*
Weight, kg, median (range)	22.1 (4.4–66.6)	16.4 (6.0–78.6)	0.025*
Malignancies, *n* (%)			0.655
ALL	41 (56.2)	38 (63.3)	
AML	14 (19.2)	11 (18.3)	
Lymphoma	10 (13.7)	4 (6.7)	
Solid tumor	3 (4.1)	4 (6.7)	
Other	5 (6.8)	3 (5.0)	

*PICC* peripherally inserted central catheter, *TCV* tunneled cuffed central venous catheter, *ALL* acute lymphocytic leukemia, *AML* acute myeloid leukemia, and *significant value

The details of catheter insertion in both groups are shown in Table 2. Regarding the number of lumens, the PICC group included 53 single lumens and 20 double lumens, while the TCV group included 42 and 17, respectively. There were no significant differences between the two groups. As for the anesthesia during catheter insertion, 59 (80.8%) patients had their PICCs inserted under sedation and 13 (17.8%) under local anesthesia. None of the patients received general anesthesia. In contrast, 60 (100%) of patients had their TCVs inserted under general anesthesia. There were no procedural complications, such as arterial injury or pneumothorax, in either of the group.

**Table 2:** Procedure details

	PICCs	TCVs	*P* value
	*n *= 73	*n* = 60	
Access vein, *n* (%)			< 0.001*
Subclavian	0 (0)	29 (48.3)	
External jugular	0 (0)	28 (46.7)	
Facial	0 (0)	2 (3.3)	
Femoral	0 (0)	1 (1.7)	
Brachial	18 (24.7)	0 (0)	
Basilic	31 (42.5)	0 (0)	
Median	14 (19.2)	0 (0)	
Dorsal hand	3 (4.1)	0 (0)	
Unknown	7 (9.6)	0 (0)	
Laterality, *n* (%)			0.514
Right	37 (50.7)	27 (45.0)	
Left	36 (49.3)	33 (55.0)	
Lumen, *n* (%)			0.533
Single	53 (72.6)	42 (70.0)	
Dual	20 (27.4)	17 (28.3)	
Unknown	0 (0)	1 (1.7)	
Anesthesia, *n* (%)			< 0.001*
General	0 (0)	60 (100)	
Sedation	59 (80.8)	0 (0)	
Local	13 (17.8)	0 (0)	
Unknown	1 (1.4)	0 (0)	
Complication rate, %	0 (0)	0 (0)	

*PICC* peripherally inserted central catheter, *TCV* tunneled cuffed central venous catheter, and *significant values

The median indwelling time was 99 days (4–413 days) for PICCs and 182 days (23–477 days) for TCVs, with the indwelling time significantly longer for TCVs (*p* < 0.001). However, the percentage of catheters that remained indwelling until the scheduled treatment was completed was not significantly different between the two groups. The incidence of catheter infection, catheter-related thrombosis, catheter occlusion, and mechanical complications that resulted in catheter removal also did not differ between the two groups (Table 3).

**Table 3:** Catheter indwelling time and the reasons of catheter removal

	PICCs	TCVs	*P* value
	*n* = 73	*n* = 60	
Total catheter service-days	9230	12,051	
Median (range)	99 (4–413)	182 (23–477)	< 0.001*
Completion, planned chemotherapy	40 (54.8)	39 (65.0)	0.233
Removal, complication			
Infection, *n* (%)	13 (17.8)	8 (13.3)	0.481
*n*/1000 catheter-days	1.41	0.66	
Thrombosis, *n* (%)	1 (1.4)	0 (0)	0.363
*n*/1000 catheter-days	0.11	0.00	
Occlusion, *n* (%)	2 (2.7)	1 (1.7)	0.678
*n*/1000 catheter-days	0.22	0.08	
Mechanical complications, *n* (%)	13 (17.8)	10 (16.7)	0.862
*n*/1000 catheter-days	1.41	0.83	
All grade adverse events, *n* (%)	29 (39.7)	19 (31.7)	0.336
*n*/1000 catheter-days	3.14	1.57	
Current use	4 (5.5)	0 (0)	
Death, with catheter functioning	0 (0)	2 (3.3)	

*PICC* peripherally inserted central catheter, *TCV* tunneled cuffed central venous catheter, *CLABSI* central-line associated blood stream infection, and *significant value

**Fig. 2:**
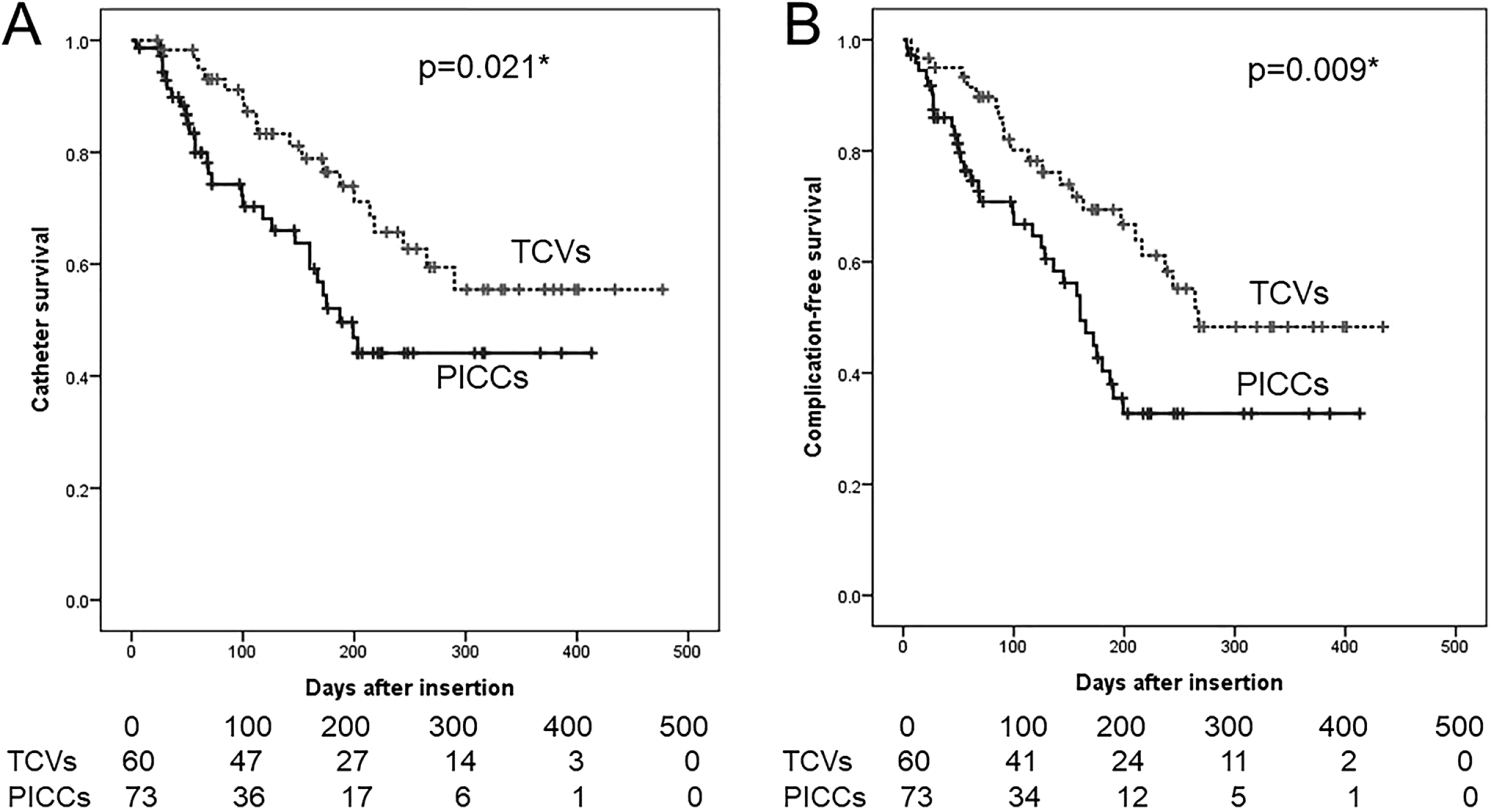
Catheter survival rate comparing TCVs and PICCs. **A** Overall catheter survival and **B** catheter survival without any complication; *PICC* peripherally inserted central catheter, *TCV* tunneled cuffed central venous catheter, and *significant values

Figure 2 shows the overall catheter survival and complication-free catheter survival for PICCs and TCVs. The overall catheter survival and complication-free catheter survival were both significantly longer for TCVs (*p* = 0.021, *p* = 0.009).

To eliminate the age difference between PICCs and TCVs, we conducted a comparison between the two groups in which patient background was corrected by propensity score matching. (Data are not shown.) For this analysis, 51 cases each of PICCs and TCVs were selected, with a median age of 5.0 years for patients with PICCs and those with TCVs. Accordingly, there were no differences in weight or height. The incidence rate of catheter infections, catheter-related thrombosis, catheter occlusion, and mechanical complications resulting in catheter removal did not differ between the two groups. Conversely, the indwelling time was significantly longer among patients with TCVs than those with PICCs (*p* < 0.001). The overall catheter survival and complication-free catheter survival were also significantly longer for TCVs, similar to the results without propensity score matching. Since propensity score matching of patient background between the two groups did not change the results of the analysis, no further matching was performed in subsequent analyses.

**Fig. 3:**
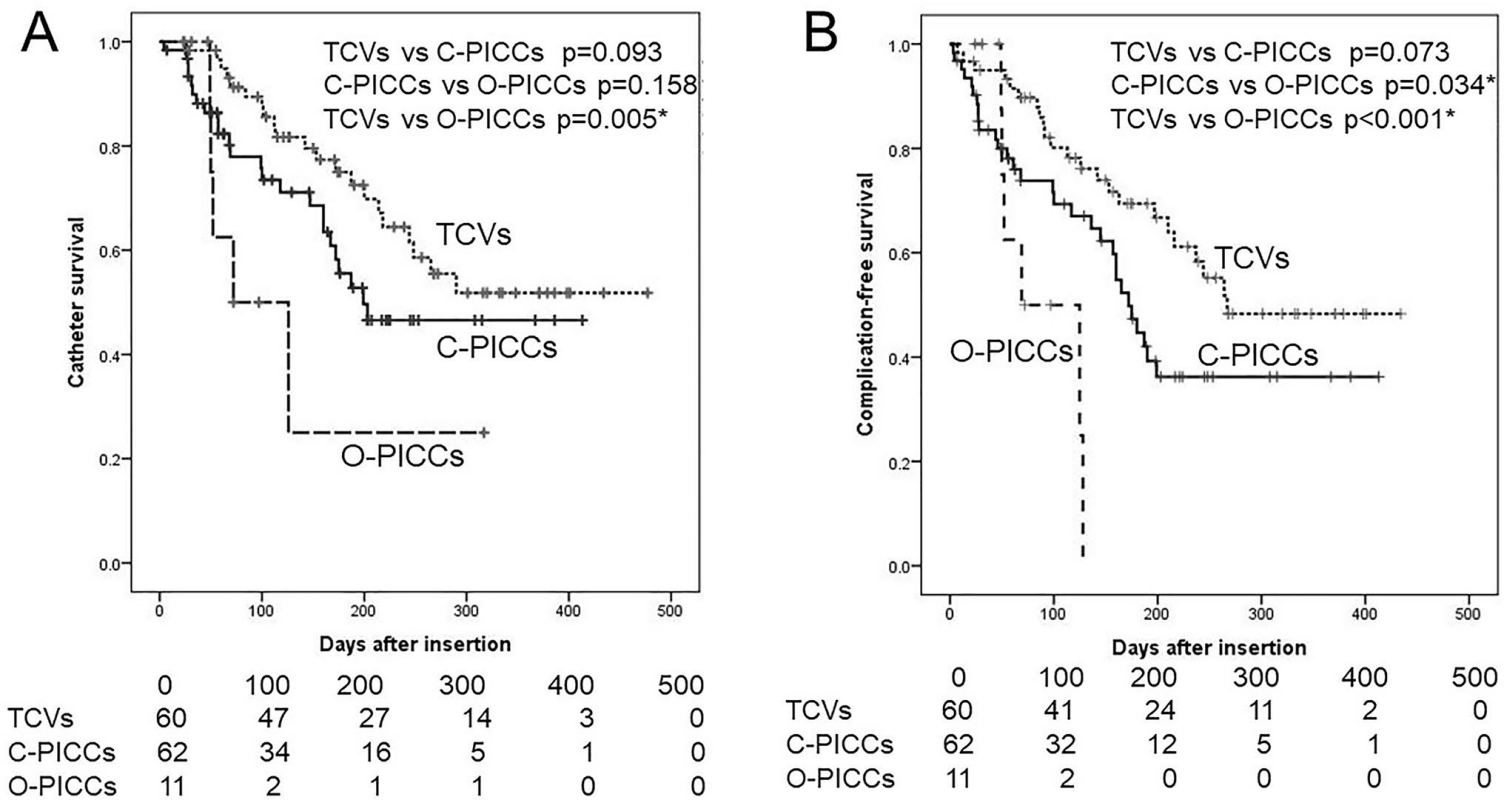
Catheter survival rate in three different central venous catheter types. **A** Overall catheter survival and **B** catheter survival without any complication. *C-PICC* closed-ended peripherally inserted central catheter, *O-PICC* open-ended peripherally inserted central catheter, *TCV* tunneled cuffed central venous catheter, and *significant values

Of the 73 PICCs in this study, 11 (15.1%) were O-PICCs and 62 (84.9%) were C-PICCs. Comparing the O-PICCs and C-PICCs, there were two cases of catheter occlusion associated with O-PICCs (18.2%) and none among the C-PICCs. As a result, catheter occlusion was significantly associated with O-PICCs (*p* = 0.001). (Data are not shown.) Comparing the overall catheter survival (A) and complication-free catheter survival (B) among the three groups (TCVs, O-PICCs, and C-PICCs), O-PICCs demonstrated significantly shorter overall catheter survival (*p* = 0.005) and complication-free catheter survival (*p* < 0. 001) than TCVs. In contrast, there were no significant differences between C-PICCs and TCVs (A: *p* = 0.093 and B: *p* = 0.073), although C-PICCs did demonstrate a trend toward a shorter survival time (Fig. 3).

Then, we compared the PICC group and the TCV group. After starting chemotherapy with TCV, PICC was inserted in five cases. Conversely, in the PICC group, no patient required TCV insertion to complete treatment, and in all cases, chemotherapy was completed with PICC alone (Fig. 1). The median age of the PICC group was 10.0 (0–16) years, which is significantly higher than the median age of the TCV group (5.0 [0–17] years; *p* = 0.037). As for the other points, there were no significant differences in sex, height, weight, or primary disease between the two groups (Table 4).

**Table 4:** Baseline demographics and patient characteristics (patient-based)

	PICC group	TCV group	*P* value
	*n* = 35	*n* = 46	
Age, year, median (range)	*1*0.0 (0–16)	5.0 (0–17)	0.037*
Male, *n* (%)	19 (54.3)	29 (63.0)	0.427
Height, cm, median (range)	135.9 (54.0–179.7)	110.0 (61.2–172.5)	0.058
Weight, kg, median (range)	27.3 (4.4–66.6)	18.5 (6.0–78.6)	0.078
Malignancies, *n* (%)			0.206
ALL	13 (37.1)	29 (63.0)	
AML	9 (25.7)	8 (17.4)	
Lymphoma	6 (17.1)	3 (6.5)	
Solid tumor	3 (8.6)	3 (6.5)	
Other	4 (11.4)	3 (6.5)	

*PICC* peripherally inserted central catheter, *TCV* tunneled cuffed central venous catheter, *ALL* acute lymphocytic leukemia, *AML* acute myeloid leukemia, and *significant value

The median number of days from diagnosis to catheter insertion was 2.0 (0–24) days for the PICC group and 9.0 (0–37) days for the TCV group. This was significantly shorter in the PICC group (*p* < 0.001). Although there was no significant difference in the total chemotherapy days between the two groups, the median number of catheters required during the treatment period was 2.0 (1–4) in the PICC group and 1.0 (1–2) in the TCV group. Hence, the median number of catheters required was significantly higher in the PICC group (*p* = 0.001). However, the median number of general anesthesia during the treatment period was 1.0 (1–2) in the TCV group and 0 in the PICC group (*p* < 0.001; Table 5).

**Table 5:** Treatment course related to catheters of each patient

	PICC group	TCV group	*P* value
	*n* = 35	*n* = 46	
Days to insertion, median (range)	2.0 (0–24)	9.0 (0–37)	< 0.001*
Total chemotherapy days (mean ± SD)	246.4 ± 122.4	254.0 ± 111.8	0.773
Total catheters, median (range)	2.0 (1–4)	1.0 (1–2)	0.001*
Number of GA, median (range)	0	1.0 (1–2)	< 0.001*

*PICC* peripherally inserted central catheter, *TCV* tunneled cuffed central venous catheter, *SD* standard deviation, *GA* general anesthesia, and *significant values

## Discussion

In this study, we found that PICC can be an alternative to TCV as a drug delivery route in pediatric patients with hematologic or oncological malignancies, since no case required TCV insertion after PICC insertion though the frequency of catheter replacement is a little higher in the PICC group.

In the adult population, the use of PICC for chemotherapy is widespread, and there are numerous reports comparing PICC and NTCV [12–15]. Evidence suggests that in the intensive care unit and for chemotherapy of hematologic tumors, PICC is associated with significantly less central-line associated blood stream infection relative to NTCV, as well as less bleeding and fewer mechanical complications at the time of insertion [13, 16].

Multiple studies have reported on the efficacy of PICC for chemotherapy in children [9, 16, 17]. However, few studies have compared PICC and TCV in pediatric patients with malignant tumors. To the best of our knowledge, only two studies have been published since 2000 [1, 18]. Both used a pressure-activated safety valve (PASV) as the PICC (C-PICC), which is different from the Groshong catheter we use. In one report, single-lumen TCVs demonstrated longer complication-free survival than double-lumen TCVs and PICCs. In the other report, complication rates did not differ between the TCV and PICC groups. The results from available studies are inconsistent. The pediatric vascular access guideline, “mini-MAGIC,” recommends the use of TCVs or TIVAPs for children over 10 kg for pediatric chemotherapy. Considering this recommendation, it is difficult to say that PICC is widely used in pediatric chemotherapy [19].

In our hospital, since 2018, PICC has been used in almost all chemotherapy in children. From our experience, PICC is minimally invasive and offers great benefits in the pediatric setting. Since current evidence is lacking, we conducted this study and compared the use of PICCs and TCVs for chemotherapy in children with hematologic and solid tumors.

Of the 133 catheters analyzed, 73 were PICCs and 60 were TCVs. The median age at insertion was significantly higher for PICCs. This may be because when PICCs were first introduced to pediatric patients undergoing chemotherapy at our hospital from 2016 to 2017, PICCs were selectively inserted in older children. Incidentally, from 2018 onward, PICCs have been used for all cases, regardless of body size. The median age was corrected for PICCs and TCVs by propensity score matching. However, the results were similar with or without propensity score matching. It has also been reported that younger age is not a risk factor for PICC complications. Consequently, propensity score matching in this study was deemed unnecessary.

As for the method of anesthesia during catheter insertion, general anesthesia was used in all cases of TCVs, while sedation was used in 59 cases (80.8%) and local anesthesia in 13 cases (17.8%) of PICCs, and general anesthesia was not required. General anesthesia in children carries risks, such as atelectasis, perioperative hypothermia, and adverse effects on brain development [20–22]. Hence, the ability to avoid general anesthesia for PICC insertion is a major advantage. Moreover, PICC insertion can be performed in patients who are at high risk for general anesthesia, especially those with solid tumors in the mediastinum and those in poor general condition due to abnormal blood cell counts or infection [23, 24].

The incidence rate of catheter infections, thrombus, occlusion, and mechanical complications did not differ between PICCs and TCVs. However, the indwelling time was significantly longer with TCVs. This may be in part due to TCVs being only inserted in patients scheduled for medium- to long-term chemotherapy originally, but after the introduction of PICC, PICC insertion was also performed in patients who were scheduled for short- to medium-term chemotherapy. In addition, because PICCs can be easily reinserted, there were several cases in which PICCs were removed at the time of discharge and reinserted at the time of readmission. These may have contributed to the shorter indwelling period for PICCs. As such, the actual difference in the indwelling time between PICCs and TCVs may be limited. In the future, a randomized controlled trial (RCT) to verify the indwelling time between PICCs and TCVs would be helpful.

In our analysis comparing O-PICCs, C-PICCs, and TCVs, there were no significant differences in catheter survival and complication-free survival between C-PICCs and TCVs. A C-PICC cannot be inserted over a guidewire and requires more refined skills for insertion, but it is less likely to cause a backflow of blood and also less susceptible to obstruction or infection than O-PICCs [12, 25, 26]. Therefore, C-PICCs as the device of choice would allow for long-term implantation similar to TCVs. If that is the case, and considering PICCs are easier to insert and remove than TCVs, we think the disadvantages of PICCs would not outweigh the advantage of avoiding general anesthesia even if the frequency of PICC replacement is slightly higher than that of TCVs.

When comparing the patients who completed treatment only with PICCs and those who only used TCVs, the PICC group experienced a significantly shorter time from diagnosis to catheter insertion compared to the TCV group. This reflects the advantage of PICCs, as they can be inserted without the need for an anesthesiologist or operating room arrangements since general anesthesia is not required. Furthermore, since the PICC can be inserted by pediatricians as well as pediatric surgeons, scheduling with pediatric surgeons is not necessary and the time to insertion may be much shorter. Early catheterization is expected to lead to an early start to treatment, and this may improve the prognosis and the general condition of the patients [16]. Simultaneously, if pediatricians can insert the catheter, it will reduce the workload of pediatric surgeons and contribute to shorter working hours of them. The results of this study, in which all patients who started chemotherapy with PICCs were able to complete treatment without subsequent insertion of TCVs, suggest that PICCs may be an effective alternative to TCVs in pediatric chemotherapy.

On the other hand, the disadvantages of PICC include the increased difficulty of insertion in young children with thin veins, the risk of accidental removal unless tightly secured because it does not adhere with a cuff like TCV, and the risk of vasculitis, which rarely occurs with TCV.

The main limitation of this study is that it is a single-center, retrospective analysis. Moreover, the historical background in which PICCs and TCVs were selected for insertion needs to be considered, because our institution has been using PICCs in almost all cases since 2018. In the future, a multi-center RCT to further investigate the efficacy of PICC comparing TCV for pediatric patients undergoing chemotherapy is warranted.

## Conclusion

Although the frequency of catheter replacement is slightly higher than that of TCVs, PICCs can be inserted easily without the need for general anesthesia and treatment can be started earlier. Taken together, PICC is an effective alternative to TCV for chemotherapy among pediatric patients with malignancies.
